# Biohydrogen production from *Euglena acus* microalgae available in Bangladesh

**DOI:** 10.1016/j.mex.2022.101976

**Published:** 2022-12-16

**Authors:** Nirendra Nath Mustafi, Md. Imran Hossain, Muhammad Faruq Ahammad, Sabrina Naz

**Affiliations:** a Department of Mechanical Engineering, Rajshahi University of Engineering &Technology, Rajshahi, 6204, Bangladesh; b Department of Botany, Rajshahi University

**Keywords:** Biohydrogen, Hydrogenase, Nitrogenase, Fossil fuel, Euglena microalgae, Hydrogen yield, Bio-photolysis of microalgae

## Abstract

Hydrogen is generally considered as an ideal non-polluting future energy carrier because it releases energy and water as a byproduct on combustion. Besides, hydrogen possesses the highest energy density on mass basis compared to any other fuel. However, hydrogen production in a sustainable and environmentally friendly way still remains a challenge. Recently, biohydrogen production from green microalgae has gained significant attention due to availability of the feedstock, which are environmentally friendly and renewable. Biohydrogen production from photosynthetic microalgae is attractive, however in the current context, it has a low yield, and an optimization of the affecting parameters including algae concentration, light intensity, culture medium, etc. is critical. In this study, biohydrogen was produced in laboratory from *Euglena acus* microalgae as it was locally available in Bangladesh.

• The effect of two different culture mediums (i.e. sulfur-rich and sulfur-deprived TAP mediums) for microalgae cultivation and biohydrogen yield were studied.
• Depending on the concentration of microalgae (50% and 75% by weight) in the medium solution ∼3 ml to 5 ml biohydrogen was obtained.

## Graphical abstract

**Figure:**
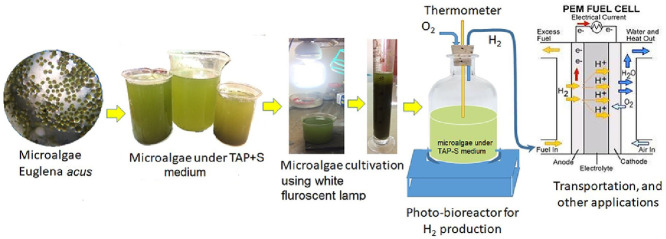

Specifications table

**Table:** 

Subject Area	Energy
More specific subject area	Hydrogen production
Method name	Bio-photolysis of microalgae
Name and reference of original method	This experimental investigation was based on the direct bio-photolysis method recommended by Sergey N. Kosourov et al. [1] who studied bio-hydrogen production from different microalgae species optimizing pH and other culture parameters under sulfur-deprived condition. This sulfur-deprived method was also used by other researchers [2]. This current study aims to employ the direct bio-photolysis method for a different microalgae species, Euglena species available locally in Rajshahi, Bangladesh.
Resource availability	N.A.

## Method details

Increased concerns about global warming resulted from the continuous emission of GHGs and depletion of oil reserves have enforced the policy makers to concentrate on renewable energies. Hydrogen is generally regarded as an ideal future energy carrier since it is easily convertible to electrical energy via fuel cells, and it releases a large quantity of energy on mass basis (141.7 MJ/Kg), without generating any air pollutant [3]. Though hydrogen is present abundantly on this planet, it exists in pure form (H_2_) at extremely low level (< 1 ppm) in the atmosphere [4]. Biohydrogen, produced biologically via microorganisms, is considered a renewable, and CO_2_-neutral energy source. Its production method is less energy intensive, less costly as well as environmentally friendly [5], [6], [7]. These microorganisms are available in nature and can produce biohydrogen through different transformation pathways: bio-photolysis, photofermentation, photocatabolism, and dark fermentation. State-of-the-art of these pathways are reviewed and described in [8,9]. Among the different pathways of biohydrogen production, bio-photolysis is considered to be the most attractive one due to its capability to generate hydrogen from water under mild environmental conditions such as at moderate temperatures and pressures (e.g., standard temperature and pressure). Biohydrogen yield from green microalgae depends on the ability of strain to supply the enzymes (i.e. hydrogenases and nitrogenases) for H_2_ metabolism and on some influencing factors such as light intensity, pH level of the medium, temperature, concentration of the substrates, etc. [10]. The design and operation of photo-bioreactors (where the reactants are put together) also play a great role in efficient biohydrogen production from algal biomass. Irrespective of the type of bioreactor, energy supply (i.e., light energy), carbohydrates, or CO_2_, are the basic requirements for biohydrogen production.

Researchers have demonstrated the potentials of H_2_ production from a variety of green microalgae such as Anabaena, Botryococcus, Chlamydomonas, Chlorococcum, Chlorella, Nostoc, Scenedesmus, Synechocystis, Tetraspora, etc. in presence of water, light/sunlight and hydrogenase and nitrogenase enzymes [11], [12], [13]. However, research studies on biohydrogen production from Euglena species particularly from *Euglena acus* is found to be very limited in the literature. In this study a microalgae, *Euglena acus* was used for biohydrogen production. Euglena is autotrophic (phototrophy) and it has chloroplasts, which enables it to fix CO_2_ into organic carbon compounds. Euglena species is found worldwide in fresh and brackish water and it has high productivity and capacity to accumulate high amounts of sugar, which make this algal biomass as an attractive substrate for biohydrogen production.

Biophotolysis involves water-splitting process in biological systems, where molecular O_2_ and H_2_ are produced in the presence of light as the energy source. Hydrogen production is generally catalyzed by the hydrogenase enzyme, which reduces protons to molecular H_2_ via reduced ferredoxin (Fd) as an electron donor [14]. Biophotolysis proceeds in two pathways: direct and indirect bio-photolysis. Direct bio-photolysis has been used in this study.

**Fig. 1:**
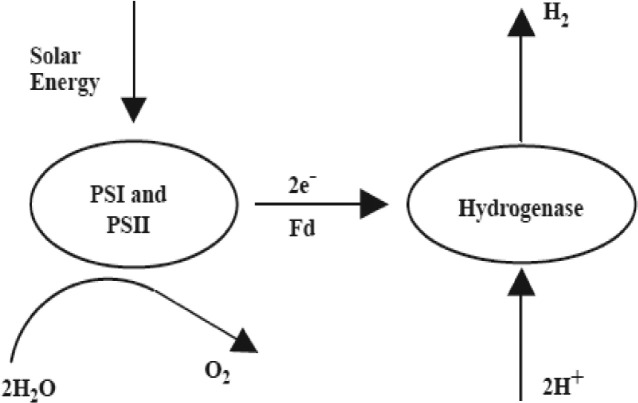
Hydrogen production by direct bio-photolysis [15].

Direct biophotolysis relies on photosystems PS-I and PS-II of green microalgae and hydrogenase enzyme (Fig. 1). Photons from the light source absorbed by PS-II (680 nm) and/or PS-I (700 nm) split water into protons, electrons/reducing equivalents, and O_2_ and reduce ferredoxin. The reduced ferredoxin eventually decreases the H_2_ evolution enzyme hydrogenase or nitrogenase directly without linking to CO_2_ fixation as an intermediate and transforms hydrogen ions to H_2_ gas in the medium by donating electrons.

Though direct bio-photolysis method has the advantages of just requiring light and water for H_2_ production, it suffers from a major issue of high sensitivity of hydrogenase to O_2_ [16]. The activity of enzyme hydrogenase is significantly affected by O_2_ concentration in the medium as it inhibits the H_2_ yield. Therefore, it is imperative to maintain the O_2_ content at a low level (<0.1%) so that H_2_ yield remains sustained [17]. Several studies have successfully demonstrated to control the O_2_ formation at low level [18], [19], [20]. Kosourov et al. [1] carried out experiments with the green algae, C. reinhardtii, to explore the effect of sulfur-deprived culture medium on O_2_ evolution and reported that this particular culture medium potentiallydecreased the activity of PS-II, which eventually reduced O_2_ formation rates and provided anaerobic condition for hydrogenase enzyme. On the other hand, the algal cells fail to survive for more than a few days in the sulfur-deprived medium [18]. This issue can be resolved easily by re-addition of sulfur to the medium, which eventually regenerates the lost algal cells for another run of H_2_ production [1]. These methodologies were employed in this current study; sulfur-rich medium was used for algal biomass cultivation and sulfur-deprived medium for H_2_ production.

**Fig. 2:**
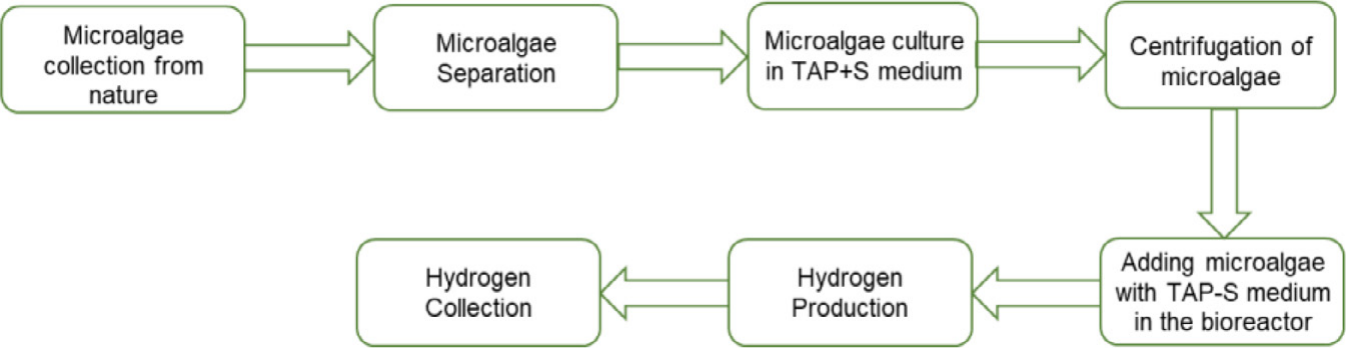
*Flow chart of hydrogen production processes from Euglena acus species microalgae*.

In the first stage, algae cells were cultivated in a medium rich in sulfur, which boosted photosynthesis reactions resulting in an enhanced algal growth rate. In the second stage, algae cells having adequate growth, were taken to a sealed container/ reactor containing another medium lacking sulfur for H_2_ production. Generally, photosynthesis process is impeded in a sulfur-deprived medium resulting in a suppression of O_2_ production even under continuous illumination [21]. Thus the O_2_ production rate by photosynthesis became lower than its consumption rate by respiration within ∼24 hours of time and algae cells became anaerobic, which led to a sustainable hydrogen production [20,^22^,23]. The two stage method of hydrogen production is shown in Fig. 2.

## Experimental procedure

### Sampling

Sampling of the target microalgae species is a crucial step for its isolation from their natural habitats. In this study, the microalgae were collected from a pond lying at 24⁰37′39′'41 N and 88.63′63’’94 E at the University of Rajshahi, Rajshahi, Bangladesh. The pond was in algal bloom condition and its pH value, TDS value and temperature were tested as 8.3, 208 mg/l, and 27.3°C respectively. The sample was collected by a plankton net (Mesh size No: 20 µm) and transferred into the glass vials or bottles and taken into the laboratory within 20 minutes in live condition. Some samples were also collected by syringe sampling, and dipping bottles into the pond directly. The algal samples were identified by the relevant literature [24]. Several abiotic factors including light intensity, water temperature, pH, and salinity were recorded during sampling in order to provide a similar environment for its cultivation in the laboratory. A global positioning system tracker was used for reference and resampling of the target microalgae species from the same location. Samples were placed in 100 ml plastic bottles containing growth medium to keep them alive and brought quickly to the laboratory.

### Isolation and dilution

Isolation of a single cell of *Euglena acus* species was done by picking a cell from the sample using a micropipette through repeated trial and error method under microscope. The single cells were then transferred to sterile droplets of water. One drop of *Euglena acus* microalgae sample was added to several drops of sterile RO water placed in the groove of a glass slide. Then confirmation of the target species was done by microscopic observation consulting with the relevant literature [24]. By repeating the same procedure microalgae washing and isolation of cells in pure form was performed.

### Preparation of media

**Fig. 3:**
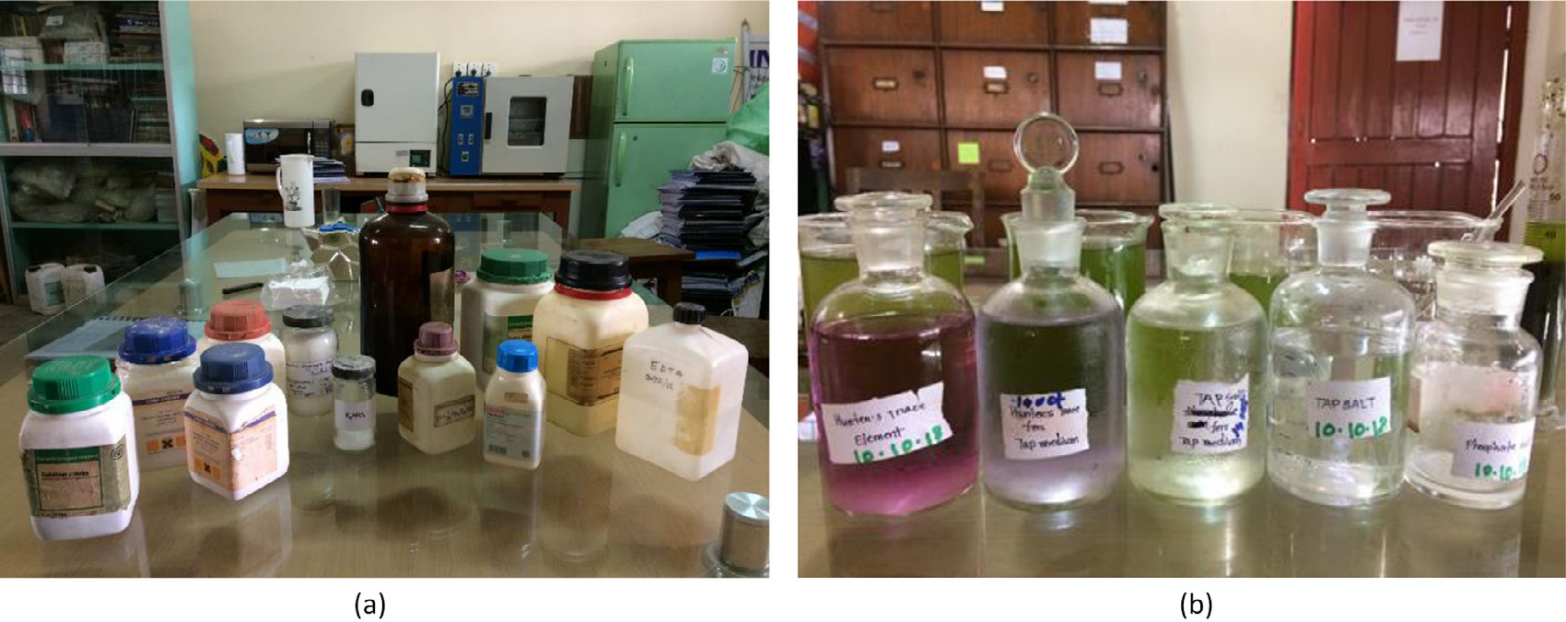
(a) Chemical reagents for microalgae culture. (b) Preparation of TAP+S and TAP-S media.

Tris-Acetate-Phosphate (TAP) has been used as a standard medium for growing green micro-alga such as C. reinhardtii in previous research studies [25]. Ethylenediamine tetraacetic acid (EDTA) content in TAP medium acts as the sole carbon source, which enhances the growth of unicellular algal cells. The list of chemical constituents of the TAP stock solution used in this study for micro-alga cultivation and H_2_ production are presented in Tables 1 and 2 respectively. All the chemicals (Fig 3a) used to prepare the growth medium were of analytical grade. After preparation, the stock solutions (Fig. 3b) were refrigerated for further use. The sulfur-rich TAP medium (TAP+S) was prepared by mixing 2.42 g of Tris, 25 ml of TAP salt, 0.375 ml of phosphate solution, 1.0 ml Hutner's Trace element and 1.0 ml of glacial acetic acid with 600 ml distilled water, as suggested in [26]. After dissolution of all the ingredients in water, the final volume of the medium was made one liter by adding additional water to it. The preparation of the sulfur-free TAP (TAP-S) medium followed the similar method except the use of equimolar chloride salts replacing sulfur salts (e.g. MgCl_2_.H_2_O in place of MgSO_4_.7H_2_O; ZnCl_2_ in place of ZnSO_4_; CuCl_2_.2H_2_O in place of CuSO_4_.5H_2_O and FeCl_2_.4H_2_O in place of FeSO_4_.7H_2_O) [26].

**Table 1:** Chemical compounds for sulfur-rich TAP stock solutions [26].

Stock solutions	Chemical Compound	Amount (g)	Water (ml)	
Tap Salt	Ammonium chloride	15±1	Dissolved in 850 ml of distilled water and finally was topped up to 1Ls	
	Magnesium sulfate heptahydrate	4±1		
	Calcium chloride dihydrate	2±0. 5		
Phosphate Solution	Dipotassium phosphate	29±1	Dissolved in 70 ml of distilled water and finally was topped up to 100 ml	
	Monopotassium phosphate	15±1		
Hunter's Trace Element	Ethylenediamine tetraacetic acid (EDTA)	5±1	25 ml	Distilled water was added to make it 1L
	Zinc sulfate heptahydrate	2±0.5	10 ml	
	Boric acid	1.5±0.5 g	20 ml	
	Manganese(II) chloride tetrahydrate	0.5±0.1	5 ml	
	Cobalt(II) chloride hexahydrate	0.15±0.01	5 ml	
	Copper sulfate pentahydrate	0.15±0.02	5 ml	
	Ammonium molybdate	0.1±0.01	5 ml	
	Ferrous sulfate heptahydrate	0.5±0.1	5 ml

**Table 2:** Chemical compounds for sulfur-free TAP stock solutions [26].

Stock solutions	Compound	Amount (g)	Water (ml)	
Tap Salt	Ammonium chloride	15±1	Dissolved in 850 ml of distilled water and finally was topped up to 1L	
	Magnesium chloride, monohydrate	4±1.0 g		
	Calcium chloride dihydrate	2±0.5		
Phosphate Solution	Dipotassium phosphate	29±1	Dissolved in 70 ml of distilled water and finally was topped up to 100 ml	
	Monopotassium phosphate	15±1		
Hunter's Trace Element	Ethylenediamine tetraacetic acid (EDTA)	5±1	25 ml	Distilled water was added to make it 1L
	Zinc chloride	1±0.5	10 ml	
	Boric acid	1.5±0.5	20 ml	
	Manganese(II) chloride tetrahydrate	0.5±0.1	5 ml	
	Cobalt(II) chloride hexahydrate	0.15±0.01	5 ml	
	Copper(II) chloride dihydrate	0.1±0.01	5 ml	
	Ammonium molybdate	0.11±0.01	5 ml	
	Ferrous chloride tetrahydrate	0.35±0.01 g	5 ml

## Cultivation of microalgae and H_2_ production

**Fig. 4:**
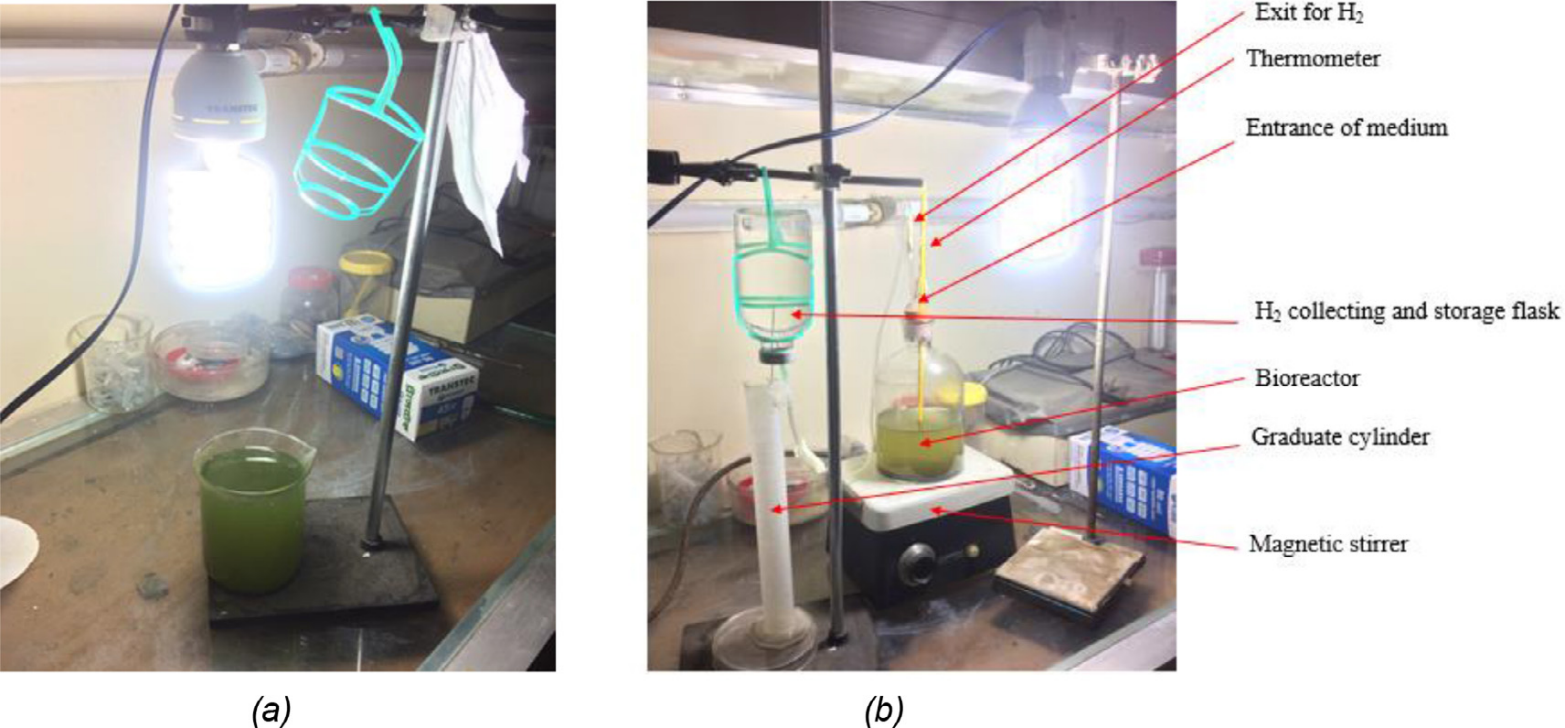
Photographs of (a) algal biomass cultivation in S-rich medium and (b) H_2_ production in S-deprived medium.

The micro-alga were diluted in 150 ml sulfur-rich TAP medium and placed in a beaker for cultivation purpose as shown in Fig. 4a. Throughout the study period, the pH of both mediums was kept approximately at 8.0. The beaker with the suspension was kept under continuous illumination by a white fluorescent light source (3000 lumen) for 4 days at room temperature (∼30°C).. During this period, the beaker was shaken a few times a day manually to prevent the culture's adhesion on the wall of the beaker. The harvesting of micro-alga after cultivation was performed by centrifugation at 3500 rpm (RCF or g (force) of 1507) for 20 min [26]. The harvested wet cell pellets were properly washed using the sulfur-free TAP solution and were placed in a 2-liter-size sealed bottle containing ∼500 ml of TAP-S medium with stirring facility. In a similar way, this TAP-S medium with the algal cells was kept under continuous illumination having the same intensity at room temperature for several days. After 4 days adequate H_2_ production rate was observed and biohydrogen yield was quantified using water displacement method (Fig. 4b). The obtained hydrogen gas was identified by simple hydrogen pop test. In this study, about 3 ml (STDEV = 0.076) to 5 ml (STDEV = 0.104) biohydrogen was obtained from 50% and 75% microalgae concentration in a medium solution of 40 ml respectively. The experimental investigation was performed for at least three times and the obtained results were averaged.

## Discussion

Biohydrogen production from microalgae is influenced by a number of factors including illumination quality and intensity, pH of the culture, ambient temperature, chemical composition of the medium (for cultivation and H_2_ production), substrate type and concentration etc. Furthermore, hydrogen yield can vary greatly for different microalgae species. Since biophotolysis is linked to photosynthesis reactions, adequate illumination by either sunlight or artificial light source is crucial for biohydrogen production. In general, an increase in light intensity increases H_2_ yield. However, at higher light intensities also enhances O_2_ production rate, which subsequently impedes H_2_ yield. The correlation between H_2_ yield and light intensity further relies on the culture age, gas phase and density of culture. As the culture grew old H_2_ production rate declined while the maximum rate of photo-production was recorded at the starting of the stationary phase [27]. However, in the current study, two concentration levels of the microalgae in the medium were investigated while the other parameters remained fixed. The higher concentration provided a higher H_2_ yield in this study. The effect of light intensity variation and culture pH on H_2_ yield is planned to be investigated in the future work.

## Declaration of Competing Interest

The authors declare that they have no known competing financial interests or personal relationships that could have appeared to influence the work reported in this paper.

## Data Availability

No data was used for the research described in the article.
